# Prevalence of Metabolic Syndrome and Its Components in Urban Cambodia: A Cross-Sectional Study

**DOI:** 10.1007/s44197-022-00053-5

**Published:** 2022-08-10

**Authors:** Miharu Tamaoki, Ikumi Honda, Keisuke Nakanishi, Sophathya Cheam, Manabu Okawada, Hisataka Sakakibara

**Affiliations:** 1 Department of Integrated Health Sciences, Graduate School of Medicine, Nagoya University, 1-1-20 Daiko-Minami, Higashi-ku, Nagoya, Aichi 461-8673 Japan; 2 Department of Pediatric, Sunrise Japan Hospital Phnom Penh, No177E, Kola Lourn Street (the Bay Road), Group2, Phurn2, Sangkat Chroy Changvar Khan Chroy Changvar, Phnom Penh, Cambodia; 3 School of Nursing, Ichinomiya Kenshin College, 5-4-1 Jougan-dori, Ichinomiya, Aichi 491-0063 Japan

**Keywords:** Metabolic syndrome, Cambodia, Urbanization, Noncommunicable diseases

## Abstract

**Background:**

The incidence of noncommunicable diseases, such as cardiovascular diseases and diabetes mellitus, is increasing in Cambodia. Urbanization and lifestyle changes due to rapid economic development have affected the components of metabolic syndrome (MetS). This study aimed to determine the prevalence of MetS, MetS components, and health status among Cambodians living in urban areas.

**Methods:**

This cross-sectional study enrolled adult Cambodians (age ≥ 20 years) who underwent a health checkup at a Japanese hospital in Phnom Penh. MetS was defined based on the harmonized diagnostic definition from the joint interim statement.

**Results:**

Among the 6090 (3174 men and 2916 women) participants who were enrolled in the study, the prevalence of MetS was 60.1% in men and 52.4% in women. The prevalence of elevated blood pressure was 73.2% in men and 65.3% in women, and was the highest MetS component in both men and women. In contrast, the lowest prevalence rates were observed for abdominal obesity (44.8%) in men and for high triglyceride levels (33.5%) in women. The MetS group showed a significantly higher proportion of patients with hypertension, diabetes, dyslipidemia, and obesity compared with the non-MetS group.

**Conclusion:**

The high prevalence of MetS in this study was attributed to urbanization, as in economically developed countries. It is necessary to explore the lifestyle habits of Cambodians that contribute to MetS and to develop preventive measures to reduce the incidence and prevalence of MetS.

## Background

The number of noncommunicable diseases (NCDs), such as cardiovascular diseases, stroke, and type 2 diabetes, is increasing remarkably in Cambodia, which is an economically developing country [1, 2]. Over the past few decades, increasing proportions of deaths caused in Cambodia have shifted from communicable to NCDs. Cardiovascular diseases were the leading cause of death in 2016, accounting for 24% of all deaths, and NCDs accounted for 64% of all deaths [3]. While treating patients with infectious diseases, such as tuberculosis, diarrhea, and HIV, it has become essential to deal with NCDs.

Metabolic syndrome (MetS) constitutes a combination of risk factors, such as elevated blood pressure (BP), dyslipidemia, hyperglycemia, and obesity, and confers a high risk for NCDs. Globally, the most common metabolic risk factors for mortality are elevated BP, followed by overweight and obesity, and elevated blood glucose levels [4]. A study published in 2010 examined the risk factors for NCDs in Cambodia and reported the prevalence of overweight and obesity as 15.4% and 1.9%, respectively, which was higher in women, whereas that of hypertension and diabetes were 11.2% and 2.9%, respectively, and were significantly higher in urban areas than in rural areas [5]. Wagner et al. reported a higher prevalence of diabetes and obesity in the urban areas of Cambodia [6]. One of the main reasons for the high prevalence of metabolic risk factors in urban areas is the change in the living environment due to urbanization, which is caused by economic development [7]. Yevgeniy et al. examined data from 173 countries and found that urbanization contributed to increased body mass index (BMI) and cholesterol levels, with a more pronounced association in low- and middle-income countries [7]. A study conducted by Maude et al. in Malaysia showed that urbanization has an impact on obesity, cholesterol levels, and hypertension [8].

In low- and middle-income countries, including Cambodia, health systems have not yet been developed to match the rapid economic growth. Medical expenses are self-paid, and there are no facilities to provide advanced medical care. Despite an increase in the middle-class and upper-class populations due to economic development, the financial burden is huge among those affected by NCDs. In addition, myocardial infarction and stroke require advanced medical care and are direct liabilities for death if untreated. As the number of people with risk factors for NCDs is high in urban areas, it is expected that the number of people with MetS, which confers multiple risk factors, will be high in urban areas. However, there are no reports on MetS in Cambodia. In this study, we examined the prevalence of MetS among Cambodians living in urban areas, which is predicted to be high, and identified the actual conditions of the target population who require intervention.

## Methods

### Data Source and Study Population

In this cross-sectional study, we used data from health checkups conducted at a Japanese private hospital, a collaborative research facility, with permission from the facility. The private hospital of this study center is located in Phnom Penh, the capital of Cambodia, and aims to provide quality medical care that conforms to Japanese standards. The hospital targets the middle-class and upper-class populations and specializes in neurosurgery, gastrointestinal surgery, and internal medicine. Most patients are from Phnom Penh or the surrounding area. According to the general population census of Cambodia 2019, Phnom Penh city and adjacent areas were categorized as urban areas [9]. An urban area was defined for this census by the National Institute of Statistics based on population size, population density, and workers in agriculture. The population of urban Cambodia in 2019 was 6.14 million, representing 39.4% of the total population, whereas that of Phnom Penh was approximately a half-million, or 8% of the total urban Cambodian population.

The participants were Cambodian citizens aged ≥ 20 years who underwent health checkups at our facility between January 2017 and December 2019. Those with complete health checkup data required for this study were included in the analysis. Individuals with any physical symptoms at the time of the health checkup were excluded.

### Anthropometric and Biochemical Measurements

Participants were instructed to consume no food or energy drinks after 9 pm, the night before visiting the hospital. We used data from electronic medical records, including blood test results [triglycerides (TG), high-density lipoprotein cholesterol (HDL-C), low-density lipoprotein cholesterol (LDL-C), fasting blood glucose (FBG)], systolic and diastolic BP, waist circumference (WC), and body mass index [BMI: body weight {kg}/height {m}^2^]). These measurements were performed by professional medical staff. After at least 5 min of rest, BP was measured on the upper arm using an automatic medical sphygmomanometer (ES-H55, Terumo Corporation, Tokyo, Japan). WC diameter is the diameter of the umbilical height measured while standing and during light exhalation. Blood tests were performed using an automated chemistry analyzer (Siemens Dimension EXL 200**,** Siemens Healthcare, Erlangen, Germany) at the hospital.

### Definition of Metabolic Syndrome

MetS was defined based on the harmonized diagnostic definition from the joint interim statement [10]. MetS was diagnosed when three of the following five symptoms were observed: (1) abdominal obesity (WC ≥ 90 cm in men and ≥ 80 cm in women), (2) elevated TG levels (≥ 150 mg/dL), or on treatment for dyslipidemia. (3) Decreased HDL-C levels (< 40 mg/dL in men, < 50 mg/dL in women, or ongoing treatment for dyslipidemia), (4) elevated BP (≥ 130 mmHg systolic BP, ≥ 85 mmHg diastolic BP), or treatment for previously diagnosed hypertension, and (5) elevated FBG level (≥ 100 mg/dL) or treatment for previously diagnosed type 2 diabetes.

### Statistical Analyses

Analyses were conducted separately for men and women, and the descriptive statistics of the participants are presented in Table 1. The Chi-square test was used to compare differences between men and women in each age group. Age-adjusted bivariate analyses were performed to compare the prevalence of MetS and MetS components in men and women and to compare the prevalence of hypertension, diabetes, and dyslipidemia between the MetS and non-MetS groups. IBM SPSS Statistics for Windows ver. 28 (IBM, Armonk, NY, USA) was used for statistical analysis, and the significance level was set at 5% (two-tailed test).

**Table 1:** Characteristics of participants

*N* = 6090	Men *N* = 3174	Women *N* = 2916
Age (years)	46.9 ± 14.5	49.3 ± 15.7
BMI	25.4 ± 3.6	23.9 ± 3.9
Waist circumference (cm)	88.5 ± 10.1	80.3 ± 10.7
Triglycerides (mg/dL)	149 (103–223)	110 (75–167)
HDL cholesterol (mg/dL)	41.9 ± 10.6	49.3 ± 12.9
LDL cholesterol (mg/dL)	128.5 ± 35.1	125.4 ± 35.7
Systolic blood pressure (mmHg)	128.6 ± 16.3	122.2 ± 19.2
Diastolic blood pressure (mmHg)	84.5 ± 11.1	77.8 ± 11.3
Fasting blood glucose (mg/dL)	110.1 ± 33.4	103.8 ± 27.6
HbA1c (%)	5.8 ± 0.98	5.8 ± 0.96

Data are presented as means ± standard deviations or medians (interquartile ranges)

*BMI* body mass index, *HDL-cholesterol* high-density lipoprotein cholesterol, *LDL-cholesterol* low-density lipoprotein cholesterol

## Results

### Characteristics of Participants

A total of 6090 (3174 [52.1%] men and 2916 [47.9%] women) participants were included in the study The statistical analysis for the characteristics of these participants is presented in Table 1.

### Prevalence of MetS

The prevalence of MetS was 60.1% in men and 52.4% in women, and the most common component of MetS was elevated BP in people of both sexes: 73.2% in men and 65.3% in women. The second most common symptom was hyperglycemia in men (66.1%) and low HDL-C in women (56.4%). In contrast, abdominal obesity for men and hypertriglyceridemia for women were the least common in 44.8% and 33.5%, respectively (Table 2).

**Table 2:** Prevalence of MetS and MetS components

	Men (*N* = 3174)	Women (*N* = 2917)	*p* value*
	% (95% CI)	% (95% CI)	
Metabolic syndrome	60.1 (58.4–61.8)	52.4 (50.6–54.2)	< 0.001
Abdominal obesity	44.8 (43.1–46.6)	53.9 (52.1–55.7)	< 0.001
Hypertriglyceridemia	52.3 (50.6–54.1)	33.5 (31.8–35.2)	< 0.001
Low HDL cholesterol	48.6 (46.8–50.3)	56.4 (54.6–58.2)	< 0.001
Elevated blood pressure	73.2 (71.6–74.7)	65.3 (63.5–67.0)	< 0.001
Elevated fasting blood glucose	66.1 (64.5–67.8)	53.3 (51.4–55.0)	< 0.001

*Age-adjusted *p* value

**Fig. 1:**
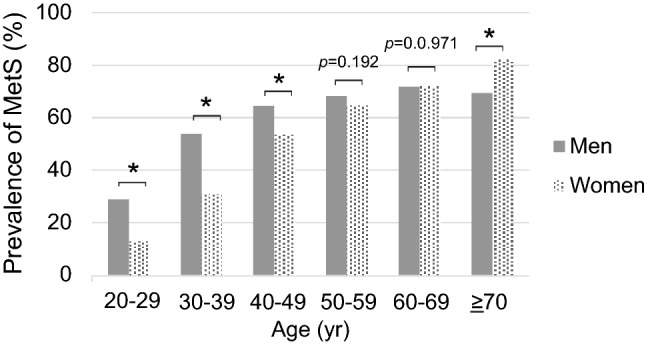
Prevalence of metabolic syndrome (MetS) by age (%)

The age-stratified prevalence of MetS showed that 54.0% of men in their 30 s had MetS. Among women in their 40 s, more than half (53.5%) had MetS. The prevalence of MetS increased with age for both men and women, with men having a significantly higher prevalence of MetS from their 20 to 40 s; however, women had a significantly higher prevalence in their 70 s (Fig. 1).

**Fig. 2:**
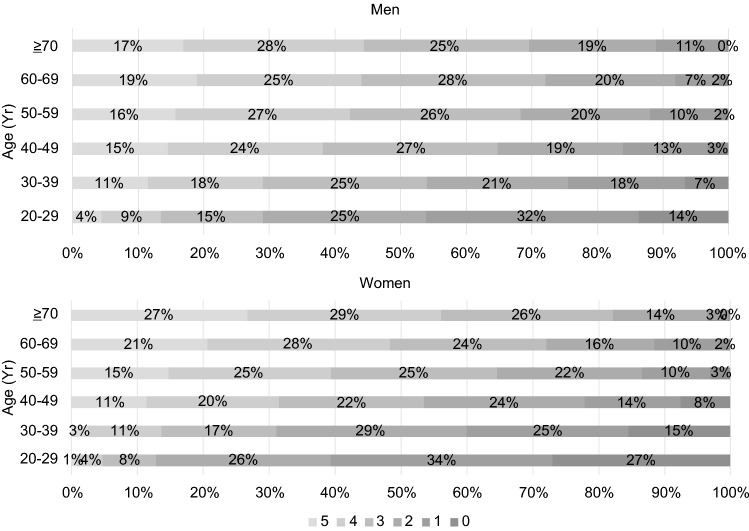
Number of metabolic syndrome components and percentage prevalence by age

Figure 2 shows the percentage of the number of components of MetS by age group. More than 90% of both men and women in their 40 s or older had at least one component of MetS. Approximately 20% of men in all age groups had two MetS components. The proportion of women with three or more MetS components increased with age.

**Fig. 3:**
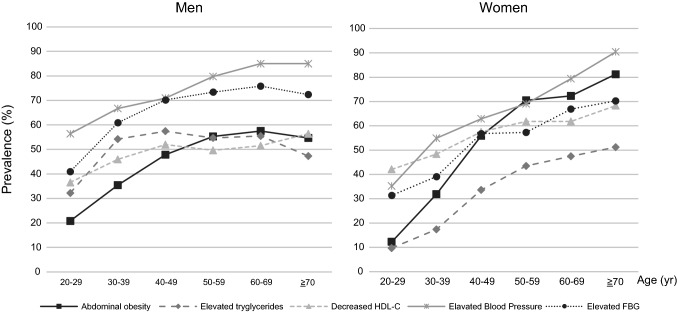
Prevalence of metabolic components by age

Figure 3 shows the prevalence of MetS components by age group. In men, elevated BP was the most common symptom in all age groups, followed by hyperglycemia. In women, except for those in their 20 s and 50 s, elevated BP was the most common symptom. In addition, abdominal obesity rises sharply in their 20 s and the highest prevalence was observed in the 50 s.

### Proportion of Patients with Hypertension, Diabetes, and Dyslipidemia and the Untreated Rate

Significantly more participants with MetS had hypertension, diabetes, or dyslipidemia than non-MetS participants. However, even among non-MetS participants, around 20% of the men had hypertension and dyslipidemia (Table 3).

**Table 3:** Prevalence of hypertension, diabetes, and dyslipidemia in the MetS or non-MetS groups

*N* (%)	Men (*N* = 3174)		*p* value*	Women (*N* = 2917)		*p* value*
	MetS (*N* = 1907)	Non-MetS (*N* = 1267)		MetS (*N* = 1528)	Non-MetS (1388)	
Hypertension^a^	891 (77.7)	255 (22.3)	< 0.001	536 (78.9)	255 (22.3)	< 0.001
Diabetes^b^	415 (87.9)	57 (12.1)	< 0.001	338 (93.1)	25 (6.9)	< 0.001
Dyslipidemia^c^	1633 (80.2)	403 (19.8)	< 0.001	979 (84.3)	182 (15.7)	< 0.001

*Age-adjusted *p* value

^a^Hypertension: systolic blood pressure ≥ 140 mmHg or diastolic blood pressure ≥ 90 mmHg

^b^Diabetes: HbA1c ≥ 6.5% or fasting blood glucose ≥ 126 mg/dL

^c^Dyslipidemia: total glyceride ≥ 150 mg/dL or HDL cholesterol < 40 mg/dL

Table 4 shows the proportions of participants with hypertension, diabetes, and dyslipidemia and the proportion of those that are untreated. Dyslipidemia was the most common type of untreated condition, accounting for 94.2% of men and 93.5% of women (Table 4).

**Table 4:** Prevalence of hypertension, diabetes, and dyslipidemia and the rate of untreated participants

*N* (%)	Men (*N* = 3174)		Women (*N* = 2917)	
Hypertension^a^	1146	(36.1)	679	(23.3)
Diabetes^b^	472	(15.3)	363	(12.8)
Dyslipidemia^c^	2036	(64.1)	1161	(39.8)
Untreated participants^d^				
Hypertension^a^	528	(46.1)	271	(39.9)
Diabetes^b^	209	(44.3)	135	(37.2)
Dyslipidemia^c^	1917	(94.2)	1085	(93.5)

^a^Hypertension: systolic blood pressure ≥ 140 mmHg or diastolic blood pressure ≥ 90 mmHg

^b^Diabetes: HbA1c ≥ 6.5% or fasting blood glucose ≥ 126 mg/dL

^c^Dyslipidemia: total glyceride ≥ 150 mg/dL or HDL cholesterol < 40 mg/dL

^d^The denominator is the number of people included in prevalence

## Discussion

To the best of our knowledge, this study is the first to identify the prevalence of MetS among Cambodians living in urban areas. The prevalence of Mets was high in urban areas, at 60.1% in men and 52.4% in women, indicating that many urban Cambodians are at high risk for NCDs.

The prevalence of MetS in this study was comparable to that reported in previous studies of urban areas in other countries: 31.3% in South Korea [11], 14.39% in China [12], 23.1% in Thailand [13], 28.4% in Indonesia [14], 34.8% in Sri Lanka [15], and 42.5% in Malaysia [16]. These countries are categorized as lower-middle and upper-middle-income countries with rapidly developing economies. Urbanization has been considered one of the reasons for the high prevalence of MetS in these countries, and its high prevalence in Cambodia may also be a result of urbanization.

The change in prevalence by age group differed between sexes. Overall, the prevalence was higher in men, although this trend was reversed in those belonging to older age groups. The prevalence of MetS in men exceeded 50% among those in their 30 s and continued to increase gradually thereafter. In recent years, it has been recognized that the prevalence of MetS is increasing among young people and has been a focus of research. Cambodia is no exception to this. This suggests a need for preventive measures starting at a young age [17, 18]. The prevalence of MetS in women increased dramatically from 31.1% in their 30 s to 53.5% in their 40 s. A Taiwanese study investigating the differences between men and women based on their development of MetS, found a higher prevalence among men until their 50 s and a higher prevalence among women after their 50 s [19]. A systematic review by Vera et al. reported that the prevalence of MetS was higher in younger men and higher in women with increasing obesity [20]. Other studies have also shown that menopause, which occurs in women in their 40 s and 50 s, is strongly associated with increased MetS [21–23]. Our results showed that the prevalence was significantly higher in men until their 40 s and significantly higher in women in their 70 s and that there was no significant difference between men and women in their 50 s and 60 s. These results, which are consistent with those of previous studies, suggest that the factors associated with differences in the prevalence of MetS by age group in urban Cambodian men and women are not specific to Cambodia, but are largely due to physical factors related to aging.

Focusing on the components of MetS revealed that they differ according to sex and age. According to the change in the number of components by sex, approximately 20% of men always had two MetS components at all age groups. However, the number of those with 0 or 1 component gradually decreased. Since three components constitute MetS, 20% of men can be interpreted as always having MetS risk; therefore, to avoid attaining MetS is important. Among women, the number of those with 0 to 2 components decreased steadily with age, indicating that the number of MetS components is steadily increasing with age.

The result of focusing on the details of MetS components showed that both men and women of all ages had a high prevalence of elevated BP. Elevated FBG level was the second most prevalent MetS component among men. Some components showed an increase in prevalence, while others showed a decrease. In contrast, the second most prevalent component for women was low HDL-C in their 30 s and 40 s, and abdominal obesity after their 50 s. The increase in the prevalence of abdominal obesity was the fastest until the 50 s. Although the prevalence was the second lowest before the 50 s, after the 50 s, it became a major factor for MetS, followed by elevated BP. The prevalence of all components continued to increase until after their 70 s. Previous studies have also shown differences in MetS components between men and women [24–26]. For example, in the United States, elevated BP was the most common factor in men and abdominal obesity in women [25]. Another study showed that, in Nigeria, women had a higher prevalence of elevated BP than men [26]. Thus, the main components differ by country as well. The difference in the prevalence of the components by age group implies that the target components to be approached for prevention may vary by sex and age. Different preventive interventions by avoiding any single metabolic risk factor depending on sex and age, may be key to preventing an increase in the incidence of MetS.

The results of a comparison of the proportion of hypertension, diabetes, and dyslipidemia between participants with and without MetS showed that significantly higher proportions of participants with MetS had hypertension, diabetes, and dyslipidemia. Our study suggests that MetS leads to a higher proportion of abnormal values, in the urban areas of Cambodia. In the non-MetS group, 20% of men had hypertension and 30% had dyslipidemia. MetS is a high-risk factor for NCDs; however, this does not imply that NCDs do not occur in the non-MetS group. This suggests that preventive interventions are necessary not only for those with MetS but also for those without MetS in urban areas.

The present study’s results for the prevalence of hypertension and diabetes and dyslipidemia were similar or higher than the prevalence of hypertension (34.7%) and diabetes (9.6%) in urban Cambodia in 2012, which was reported by Wagner et al. in 2018 [6]. Moreover, the prevalence was higher than the prevalence of hypertension (29.7%) and diabetes under treatment (5.6%) in urban Cambodia reported by the STEPS survey in 2010 [5]. Urbanization in Cambodia has spread rapidly, more than doubling from 19.5% in 2009 to 39.5% in 2019 [9]. In particular, Phnom Penh and its suburbs have been developed and are growing rapidly. Previous studies have shown an association between the degree of urbanization and risk factors for NCDs [27]. The increasing trend in the prevalence of hypertension and diabetes in urban Cambodia, based on the results of this study and previous studies, suggests that although the study participants were from a single institution, the results represent urban results and reflect the progression of urbanization.

Furthermore, by determining the proportion of untreated people with hypertension, diabetes, and dyslipidemia, we found that more than 40% of men and 30% of women with hypertension and diabetes were untreated, and more than 90% of people of both sexes with dyslipidemia were untreated. The STEPS survey reported an untreated hypertension rate of 72.2% in Urban Cambodia and that 37.2% and 71.2% of urban participants had never had their BP and blood glucose levels measured, respectively, although the percentages were lower than in the rural areas [5]. Thus, untreated rates may be similarly high for diabetes and dyslipidemia, although these were not reported in the STEPS survey. Another report on untreated rates reported a high untreated rate similar to that in Cambodia, as 42.3% of urban participants in India aged ≥ 45 years had undiagnosed hypertension [28]. Moreover, data reported from China indicate that 49.0% and 25.2% of the participants were unaware of their hypertension and were untreated, respectively, and that factors associated with untreated hypertension included age and education level [29]. The relatively low untreated rate in this study, compared with that in the STEPS survey in 2010, is attributable to a greater concern of the participants about their health than the general urban population, which may have led to a higher proportion of our participants being diagnosed and treated. Health checkups are common in developed countries in the primary prevention of disease. However, health checkups are not common and widespread in Cambodia and in other developing countries. Thus, a high untreated rate and unawareness of disease could lead to the unintentional development and progression of NCDs. Furthermore, the high untreated rate in developing countries may contribute to the increase in NCDs unless preventive measures are implemented. This study identified a large number of untreated participants in Cambodia who need treatment, suggesting the necessity for early intervention.

The strength of this study is that it presents the prevalence of MetS and its components in an urban area of Cambodia for the first time. However, there is a possibility of sampling bias because the participants were from a single institution, and health checkups are not common in Cambodia; therefore, it is likely that these population has health concerns or are relatively interested in health. Thus, the results of this study have a limitation in representing urban Cambodia as a whole. However, the results show the real-world prevalence of NCDs in urban Cambodia that is strongly affected by urbanization, and this study provides an important indication of the need for MetS prevention.

## Conclusion

The prevalence of MetS among Cambodians living in urban areas is 60.1% in men and 52.4% in women. This high prevalence is attributable to the influence of urbanization, similar to that in economically developed countries. The prevalence of MetS and its components differed according to sex and age. Our study provides valuable information for Cambodia, where no reports of MetS have been presented yet. Further studies are needed to explore the factors necessary for prevention and develop preventive measures for early intervention.

## Data Availability

The datasets generated during and/or analyzed during the current study are available from the corresponding author on reasonable request.
